# A Treatise on Hernia

**Published:** 1817-11

**Authors:** 


*A Treatise on Hernia; by* ANTONIO SCARPA, *of Pavia.* Translated by J. H. WISHART, M. R. C. S. &c. One Vol. Octavo. pp. 548; with many plates.

CONSIDERING the slowness and uncertainty with which medical publications circulate among the inferior orders of the profession, we shall make no apology for introducing a concise Analytical Sketch of the more interesting and practical features of the celebrated Pavian Professor's work on Hernia.

The first Memoir is on Inguinal and Scrotal Hernia. We shall pass over the mere anatomical sections of this memoir, and begin our Analysis at p. 42, on "THE IMMEDIATE CAUSE OF HERNIA." Many celebrated modern surgeons adopt the opinion of Morgagni and others, that the principal cause of inguinal hernia, in particular, "consists in the relaxation and elongation of the mesentery" in consequence of which the whole mass of intestines presses constantly on the inguinal ring, and at last forces a passage out of the abdomen.

"In a state of perfect health, there are two sets of forces, in equilibrium with each other; the one, the force of pressure of the abdominal viscera against the parietes of the abdomen, and the other of re-action of these parietes upon the contained viscera. If this reciprocity of forces were the same in all persons, and in all circumstances of life, herniæ would never occur; or if from disease the abdominal parietes always yielded equably at every point of the circumference of the abdomen to the impulse of the contained viscera, there would indeed be persons with large bellies, but never affected with hernia, properly so called. Although the liver, the spleen, and the stomach are provided with ligaments, such means would be only a very weak support to retain these viscera in their situation, if there was not in addition to this a constant fulness, a continual pressure produced by the reciprocal action of the containing and contained parts of the abdomen, which causes every viscus to contribute to keep the contiguous one in its proper position. But there are some points of the circumference of the abdomen naturally less resistant than others, especially the space extending from the superior and anterior spine of the ilium to the pubes. These points are necessarily less capable than the others of re-acting against the succession, sometimes too violent, with which the compressed abdominal viscera are forced outwards. And these parts of the abdomen are in some individuals still less resistant than in others, on account of congenital relaxation and slenderness of make, or of many various external and internal debilitating causes. Whenever, therefore, the force of the pressure upon the viscera is unusually increased, or the natural resistance is much diminished in some point of the abdomen, which is as much as to say, when there is an alteration of the just equilibrium between the two orders of forces above mentioned, it must follow, from mechanical necessity, that the sum of the forces exercised by the abdominal muscles, by the diaphragm and by the elevator muscles of the anus, will be directed and concentrated wholly towards the least resisting point of the abdomen, against which point that viscus will be pushed, which, from its vicinity or its mobility, is the most disposed to be thrust towards the least resisting place of the abdomen. If this viscus be a loop of intestine, it will necessarily follow, that the concentration of the compressing muscular forces will act simultaneously upon the intestine, and upon the corresponding portion of mesentery, which portion of membrane will be similarly, and at the same time, relaxed and elongated in proportion as the intestine is protruded out of the abdomen to form a hernia. In those cases, in which the resistance of the abdominal parietes, to be overcome by the compressed viscera, is not very considerable, as in congenital hernia, in which the hernial sac is already prepared for receiving the protruded gut, the loop of intestine quickly displaces and draws along with it the corresponding portion of mesentery. On the contrary, in the common inguiual hernia, from quite opposite reasons, this does not take place with equal facility, as in the congenital ; and in general, on the first removal of the equilibrium between the abovementioned forces, there is not formed a hernia properly so called, but there appears only a slight fulness of the groin for a certain space, from the superior and anterior spine of the ilium towards the inguinal ring. When the protruded intestine has passed the inguinal ring, the stretching of the mesentery and increase of the hernia make rapid and simultaneous progress." p. 46.

Many interesting observations follow, on the situation of the hernial sac, the changes which the cremaster muscle undergoes in old hernia, the changes in the inguinal ring, &c. for which we must refer to the work itself. The following case of large hydrocele, where the spermatic artery was wounded, we shall extract.

" On the 3d of February, Angelo Maria Rossi, of the district of Cavandone, applied to me to be punctured for a double hydrocele, which he had had for five years. I placed the patient in the proper posture, and having laid hold of the scrotum from below, I compressed the fluid, without being able to feel the testicle at its attachment to the vaginal coat. I took the *trocar* and introduced it a little obliquely at the lower part, to give a greater declivity to the water. On withdrawing the *trocar*, blood immediately issued, mixed with an almost gelatinous fluid, and I was repeatedly obliged to clear the canula, as several fibres resembling melted down cellular substance collected in it, and prevented the passage of the water, blood always continuing to be discharged, mixed with the fluid. Having emptied the tumour, as much as possible, I withdrew the canula, not considering the discharge of blood of any importance, supposing that I had wounded some small cutaneous vessel in making the puncture. I applied some lint, with a suspensory bandage, and the patient departed to return home. After having proceeded a quarter of an hour on his journey, he observed that the tumour began again to swell, and he felt, as he expressed it, like the beating of his heart in it. Having returned back, he again came to me; and on examining the tumour, I found it much enlarged, and blood oozed out by the aperture formed by the canula, with which the whole dressings were soaked. I laid hold of the integuments round the opening with two fingers, and I kept them compressed for some time, to endeavour to stop the blood, always supposing it to be some vessel of the scrotum which had been wounded. But the tumour continued to increase, and a pulsation was felt in it; I then resolved to open the tumour, to see in what manner the hæmorrhage could be stopped. With a bistoury, I began the incision from the opening made by the canula upwards towards the ring. The moment the incision was made, a great quantity of blood gushed out, which I knew to be arterial, and I saw that it flowed *per saltum*, from the upper part near to the ring. I extended the incision to the ring, and instantly a large arterial vessel presented itself to me, which poured out blood copiously; and having laid hold of it with the tenaculum, I tied it; after which the hæmorrhage stopped, except the blood discharged from the incision. As I did not know how this accident had happened, I laid the testicle completely bare, to ascertain, if it were possible, that the spermatic artery had been wounded by the *trocar;* which, in fact, I found to be the case, the testicle only remaining attached by a small thread, which did not pulsate when taken between the fingers; and I farther discovered, that the cord was divided into two parts from the ring downwards, without its usual sheath. Considering, therefore, that the testicle could not have lived without the nourishment of the blood, the spermatic artery being completely divided, I removed it. I applied the necessary dressings, and the cure going on regularly, the patient soon recovered, having still the other hydrocele, which I will never venture to puncture, in order to avoid the accident which happened in the former." P. 78.

From the second Memoir, we shall condense some of the Professor's observations on the operation for incarcerated scrotal hernia.

When the tumour is of moderate size, the incision of the integuments may be a little on either side of the longitudinal axis; but in old and large hernia, the incision should be exactly in the middle of the tumour; because, in the latter case, the vessels composing the spermatic cord are frequently separated and forced towards the sides of the hernia. This consideration should also prevent us from cutting away the sides of the hernial sac after replacing the viscera. Our author advises great caution in making the opening into the hernial sac, in consequence of the uncertainty as to the layers of condensed cellular membrane covering it.

After opening the sac, the principal object is to free the viscera from strangulation with safety and expedition. The principal stricture is found, not so much in the ring, as in the neck of the sac. In opening this, the Professor asserts, that the epigastric artery has not very unfrequently been wounded, and death thereby occasioned. To guard against this fatal accident, Prof. Scarpa recommends "the division of the ring, and of the neck of the hernial sac, in a line parallel with the linea alba, so that the incision may form a right angle with the horizontal ramus of the pubes; and this incision should be always small, and not caused by those long cuts very improperly practised by some surgeons." 129.

As a regular analysis of an elementary work like this, is quite out of the question, we must content ourselves with a few specimens from the different Memoirs. From the fourth, we shall make a few extracts; and first, on the proper time for operating in hernia.

"But whether we treat of the *acute* or *slow* strangulation, surgery is still defective in precise and accurate rules, to enable us to determine, in individual cases of incarcerated hernia, to what period the operation for hernia may, with safety, be deferred, and when it ought to be performed immediately, or soon after the incarceration. I have no doubt, however, that with the assistance of numerous observations, carefully made and compared together, the art is likely to make considerable progress on this very important point of the treatment of hernia. In the mean time, I think it will be of advantage, to relate here the facts which have occurred to me, with regard to the marks of the one or other case; which, in the course of my practice, have appeared to me the least fallacious, and to show that, in general, the operation is too long delayed: and this is the reason, in my opinion, why several of the greatest masters of the art are more unfortunate in the practice of this operation, while many other surgeons, very much inferior in learning and manual dexterity, but prompt in operating, almost always obtain favourable results.

"In the first place, when the strangulation is so slow and moderate, that it intercepts the course of the feculent matter, but does not compress deeply the substance of the intestine, the hernia is swelled, but not painful; the abdomen is soft, and although distended, bears pressure. The patient complains of general restlessness, of belching of wind, of dryness of the tongue and fauces, and of loathing of the stomach, resembling nausea. After twenty-four or thirty hours have elapsed in this state, vomiting comes on, dryness of the skin, fever, with sometimes a hard and slow, sometimes a soft and frequent pulse. On the contrary, in young and robust persons, when the strangulation of the intestine, at its first appearance, is so violent as not only to intercept the course of the feculent matter, but likewise to compress deeply the substance of the intestine, and actually to strangulate it, vomiting and general anxiety arise almost at the same moment the strangulation of the bowel takes place, and the efforts of vomiting continue, although the stomach be quite empty. Further, if the patients are young and stout, the hernia not only very soon becomes painful, but the whole abdomen also is sensible to the most gentle touch, an undoubted and dreadful mark, that there is a threatening of an attack of inflammation, or that it has already spread over the whole circumference of the peritonæum; hiccup begins, the pulse is hard, contracted, quick, with extreme depression of body and mind of the patient, the more marked if he was naturally lively and strong. Soon after, both in the *slow* and *acute* strangulation, when the loss of vitality of the strangulated intestine is about to supervene, or has already come on, the patient says that he feels relieved from the excessive pains which he felt in the hernia and in the abdomen, and from the continual tendency to vomiting, which had so much distressed him from the beginning of the disease. But, instead of these distressing symptoms, he is affected by others still more violent and formidable; such as hiccup more urgent than before, which is a fatal symptom, especially in old persons; a cold sweat over the whole surface of the body, so that the skin is less warm than that of a person who has recently expired. The pulse is small, irregular, tremulous; the countenance ghastly; the functions of the brain disturbed; the skin covering the hernia is tinged of a red colour, with livid streaks. This redness of the skin of the scrotum, conjoined with the preceding symptoms, constantly marks the near approach of sphacelus of the parts contained in hernia; and if the tumour yields on pressure, giving a feel of crepitation, the sphacelus of the protruded parts is already complete. I am aware, that the intestine is sometimes found livid and black, although the symptoms of strangulation have been *slow*, and the operation performed in very good time. I could adduce several such facts; but whenever I have examined the subject attentively, in similar circumstances,

I have found that this livid and blackish colour of the intestine was not produced by inflammation, and still less by gangrene, but was rather the effect of *sugillation*, which may take place although the intestine has not been deeply constricted, but merely compressed. This is demonstrated by the *sugillated* or *ecchymosed*, as it is called, and blackish intestine, preserving, nevertheless, its natural consistence and globular shape, and aptitude to resume its functions, after being replaced in the abdomen, while that which is deprived of its vitality gangrened, and approaching to sphacelation, besides exhaling a cadaverous fœtor on the first opening of the hernial sac, is likewise depressed, flaccid, and the slightest touch of the fingers of the operator strips it of its external coat, as it is black and hard, or, as some call it *hepatised;* in which circumstances, it is no longer in a state to be replaced. These unfortunate circumstances ought not, however, to be considered as contra-indicating the operation; for if even the mortification of the intestine has begun, the operation would always be the only means of saving the patient, in so far as the division of the intestine facilitates the discharge of the fæces, diminishes the painful tension of the abdomen, and promotes the separation of the gangrenous parts." p. 295.

Our limits unfortunately prohibit us from being as diffuse, in our analysis of this work, as we could wish. We shall, therefore, conclude our Review of Professor Scarpa's work, by condensing his remarks on cases of *adventitious* umbilical hernia in infants. He considers the principal means of cure of these herniæ, as of all others, in general, to consist in replacing the protruded parts as quickly as possible, and in keeping them constantly replaced by means of suitable compression. In cases of recent umbilical hernia in infants, this is easily performed, for the protruded viscus is the intestine, free from all adhesion internally with the peritonæum, and externally with the hernial sac; in these cases also the umbilical ring still preserves a certain degree of tendency to contract, which the pressure employed, and preservation of the supine posture, facilitates. A belt of linen, with a pad of the convexity of half a nutmeg, will keep the umbilical tumour perfectly well replaced in these children. It should be sufficiently elevated on the abdomen by means of one or two compresses, so that the points of compression may rest exactly upon the umbilicus and on the spine, and, consequently, press as little as possible on the *sides* of the abdomen, while it holds the viscera in their proper situation. This pad, in the form of a button, has been proved by experience to be preferable to the flat compressor; because, by pressing the protruded viscera deeply and completely into the abdomen, it places the skin in firm contact with the aponeurotic margin of the opening, without at all preventing the contraction of the umbilical ring. Professor Scarpa describes a very simple and efficacious form of compression after the protruded parts are returned.

"A piece of fine linen is to be applied over the depression of the umbilicus, and over it a button or pad, which is to be kept *in situ* by means of some strips of plaster, crossed in the figure of the letter X. From the centre of the pad, a thread passes out through the middle of one or more compresses fixed to the circular bandage. A belt of double linen or fustian is passed round the abdomen, and is drawn moderately tight till it returns over the compress, to which it is fastened by two tapes on each side."

In children more advanced in years, the *scapulary* and *thigh-strap* should be added to this bandage. It may also be made elastic by the addition of two elastic springs, corresponding to one-third part of the whole length of the belt. Our author thinks, that the operation of ligature in umbilical hernia is attended by violent and dangerous symptoms, and that it does not produce a radical cure, without the assistance of compression continued for several months after the wound is cicatrized. On the contrary, he says, compression alone, for three or four months, will accomplish the radical cure in infants, without any danger.

From the foregoing analytical specimens of this interesting work, our readers will perceive that it deserves a place by the side of Cooper and Lawrence in every surgical library. Mr. Wishart has executed the task of translation with his usual ability and taste.

